# Influence of strain, age, origin, and anesthesia on the cardioprotective efficacy by local and remote ischemic conditioning in an ex vivo rat model

**DOI:** 10.14814/phy2.14810

**Published:** 2021-04-04

**Authors:** Thomas Ravn Lassen, Marie Vognstoft Hjortbak, Marie Hauerslev, Pernille Tilma Tonnesen, Steen Buus Kristiansen, Rebekka Vibjerg Jensen, Hans Erik Bøtker

**Affiliations:** ^1^ Department of Cardiology Aarhus University Hospital Aarhus N Denmark; ^2^ Department of Clinical Medicine Aarhus University Aarhus N Denmark

**Keywords:** cardioprotection, heart, ischemic conditioning, isolated heart preparation

## Abstract

**Background:**

Local ischemic preconditioning (IPC) and remote ischemic conditioning (RIC) induced by brief periods of ischemia and reperfusion protect against ischemia‐reperfusion injury.

**Methods:**

We studied the sensitivity to IR‐injury and the influence of strain, age, supplier, and anesthesia upon the efficacy of IPC and RIC in 7‐ and 16‐weeks‐old Sprague‐Dawley and Wistar rats from three different suppliers. The influence of sedation with a hypnorm and midazolam mixture (rodent mixture) and pentobarbiturate was compared.

**Results:**

IPC attenuated infarct size in both 7‐weeks‐old Sprague–Dawley (48.4 ± 17.7% vs. 20.3 ± 6.9, *p* < 0.001) and 7‐weeks‐old Wistar (55.6 ± 10.9% vs. 26.8 ± 5.0%, *p* < 0.001) rats. Infarct size was larger in 16‐weeks‐old Sprague–Dawley rats, however, IPC still lowered infarct size (78.8 ± 9.2% vs. 58.3 ± 12.3%, *p* < 0.01). RIC reduced infarct sizes in 7‐weeks‐old Sprague–Dawley (75.3 ± 11.8% vs. 58.6 ± 8.9%, *p* < 0.05), but not in 7‐weeks‐old Wistar rats (31.7 ± 17.6% and 24.0 ± 12.6%, *p* = 0.2). In 16‐weeks‐old Sprague–Dawley rats, RIC did not induce protection (76.4 ± 5.5% and 73.2 ± 14.7%, *p* = 0.6). However, RIC induced protection in 16‐weeks‐old Wistar rats (45.2 ± 8.5% vs. 14.7 ± 10.8%, *p* < 0.001). RIC did not reduce infarct size in 7‐weeks‐old Sprague–Dawley rats from Charles River (62.0 ± 13.5% and 69.4 ± 10.4% *p* = 0.3) or 16‐weeks‐old Wistar rats from Janvier (50.7 ± 11.3 and 49.2 ± 16.2, *p* = 0.8). There was no difference between sedation with rodent mixture or pentobarbiturate.

**Conclusion:**

The cardioprotective effect of IPC is consistent across rat strains independent of age, strain, and supplier. RIC seems to be less reproducible, but still yields protection across different rat strains. However, age, animal supplier, and anesthetics may modulate the sensitivity of IR‐injury and the response to RIC.

## Graphical abstract

Cardioprotection by ischemic conditioning remains a promising treatment strategy, however this study substantiates the importance of unifying basic methods in preclinical studies of cardioprotection.

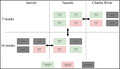

## 1 INTRODUCTION

Local ischemic preconditioning (IPC) by brief cycles of ischemia and reperfusion (IR), prior to prolonged ischemia, reduces infarct size substantially (Murry et al., 1986). The cardioprotective stimulus can also be employed in a distant organ, such as the upper arm (Kharbanda et al., 2002; Przyklenk et al., 1993). Hence, the concept of ischemic conditioning has evolved into the more clinically applicable approach, remote ischemic conditioning (RIC). IPC seems to exert stronger protection than RIC (Botker et al., 2018a). Nevertheless, RIC is useful in unpredictable ischemia including acute myocardial infarction and stroke as it can be applied before, during or even after the ischemic event (Przyklenk et al., 1993; Schmidt et al., 2007; Zhao et al., 2003). Despite substantial and promising experimental data, translation of ischemic conditioning to the clinic has proven challenging (Hausenloy et al., 2019; Heusch, 2017).

To overcome these challenges, stable and reliable ex vivo methods are pivotal for studying the underlying mechanism of cardioprotection by IPC and RIC (Botker, Hausenloy, et al., 2018). Different species have varying sensitivity to IR‐injury and responsiveness to cardioprotective interventions (Galinanes & Hearse, 1990). Even different strains within the same species, such as rats, may have different sensitivities to IR‐injury (Baker et al., 2000). The infarct reducing effects of IPC and RIC have been validated in several rodent species like mice, rats, and rabbits (Hauerslev et al., 2018; Jensen et al., 2012; Johnsen et al., 2016) and in large animals including pigs and dogs (Murry et al., 1986; Schmidt et al., 2007). IPC and RIC are consistently inducible in all rodent and pig models in vivo (Bromage et al., 2017). RIC significantly reduces infarct size in in vivo models of myocardial IR‐injury and heterogeneity between studies does not seem to be explained by a number of experimental variables tested (Bromage et al., 2017). We have experienced that not only infarct size, but also the infarct size reducing the capacity of RIC seems highly variable in ex vivo rat models depending on strain, age, origin, and anesthesia despite uniform experimental conditions.

Therefore, the aim of the present study was to detail the influence of strain, supplier, age, and choice of anesthesia on the cardioprotective efficacy of IPC and RIC in the Langendorff‐perfusion setup using a rat model.

## 2 METHODS

### 2.1 Animals and study design

We used male 7 and 16‐weeks‐old Wistar and Sprague–Dawley rats and included a total of 183 animals. The experiments conformed to Danish law (act. no. 1306 of 23/11/2007) and institutional guidelines for animal research and were approved by the Danish ethical research committee (M‐2016‐218‐16). Animals were acquired and kept at a constant temperature of 23°C with a 12‐h light‐dark cycle and allowed unlimited access to chow and water. Animals were acquired one week prior to the experiments and reached the specified age at the time of the experiments.

**FIGURE 1:**
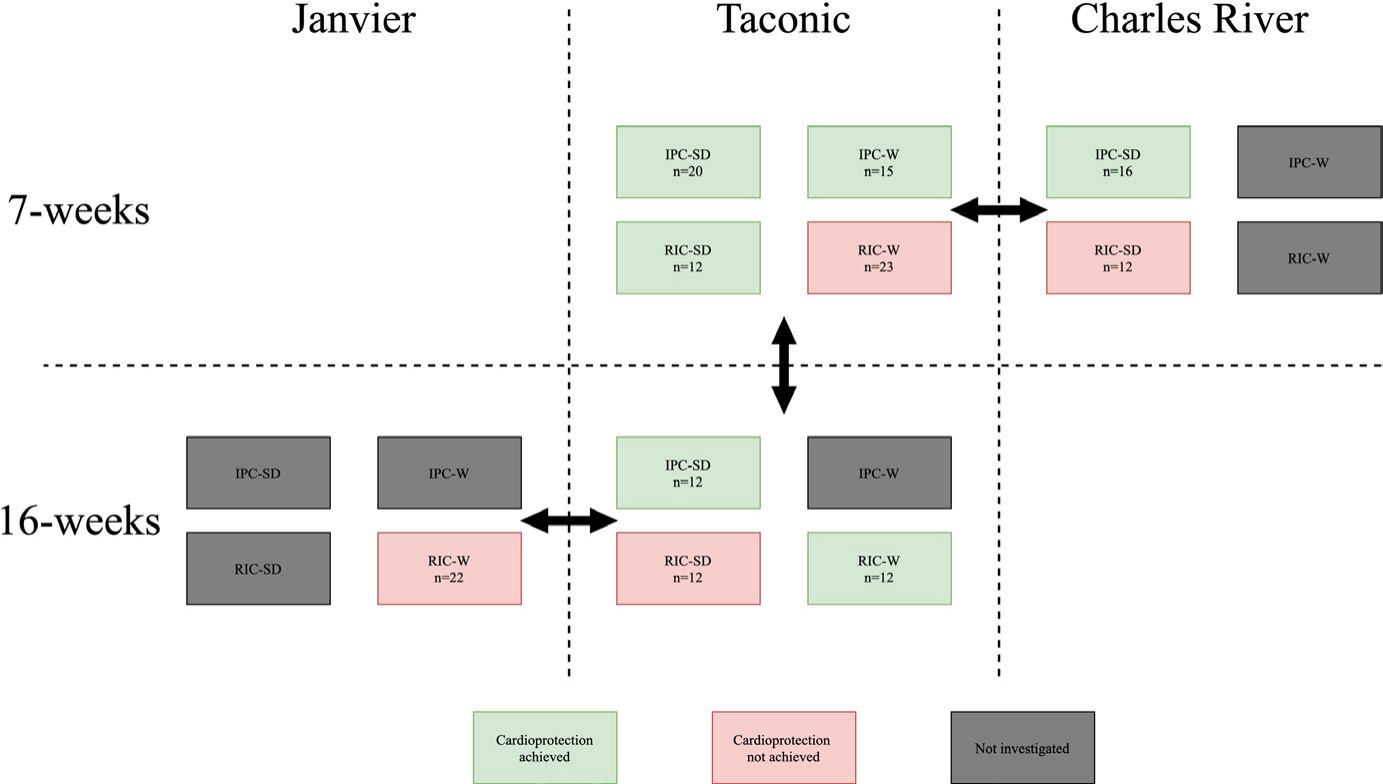
Graphical representation of the outcome interventions by IPC and RIC. Arrows indicate comparison between the three different suppliers, Janvier, Taconic, and Charles River and the comparison between the two age groups (7‐ and 16‐weeks) in rats from Taconic. The colors indicate if we found a significant difference between the intervention and control groups. IPC‐SD, Ischemic preconditioning in Sprague–Dawley rats; IPC‐W, Ischemic preconditioning in Wistar rats; RIC‐SD, Remote ischemic conditioning in Sprague–Dawley rats; RIC‐W, Remote ischemic conditioning in Wistar rats

We investigated whether infarct size reduction by IPC and RIC was achievable in 7 and 16‐week‐old rats from Taconic (Taconic), Charles River (Charles River, Jackson Laboratory), and Janvier (Janvier), such that we could evaluate the influence of strain, age, and origin by comparisons as presented in Figure 1. Additionally, we evaluated the effect of sedation with pentobarbiturate against a hypnorm and midazolam mixture (rodent mixture) in 7‐weeks‐old Sprague–Dawley rats from Taconic (not shown in Figure 1).

### 2.2 Ex vivo perfusion model

**FIGURE 2:**
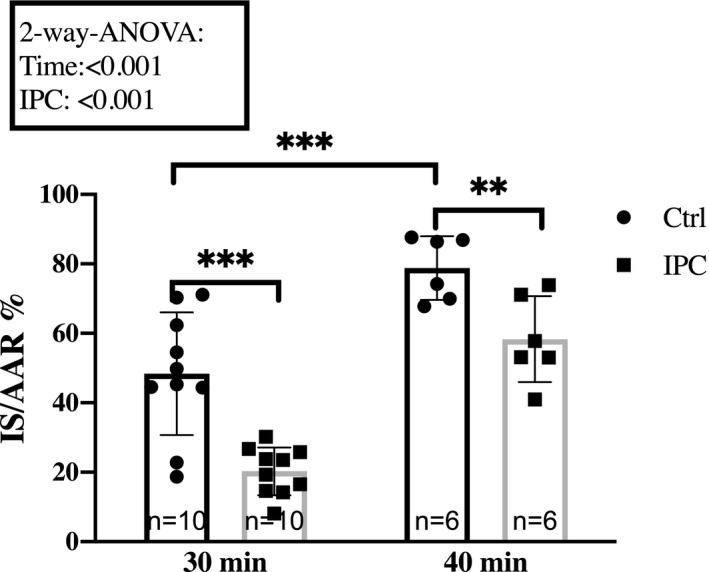
Effect of ischemia time. Response to 30 min and 40 min ischemia in hearts from 7‐weeks‐old and 16‐week‐old Sprague–Dawley rats, respectively, sedated with rodent mixture. Mean ± SD. *p < 0.05, **p < 0.01 and ***p < 0.001. Ctrl, Control; IPC, Ischemic preconditioning; IS/AAR, infarct size/Area at risk; RIC, Remote ischemic conditioning

To investigate the cardioprotective effect of IPC and RIC, we used the Langendorff perfusion model. Rats were anesthetized, intubated, and connected to a ventilator. Through a thoracotomy, the heart was dissected from the surrounding structures and cannulated in‐situ before being mounted in the isolated heart apparatus. The hearts were perfused with Krebs–Henseleit (KH) buffer (composition in mM: NaCl 118.5, KCl 4.7, NaHCO_3_ 25.0, glucosemonohydrate 11.0, MgSO_4_·7H_2_O 1.2, CaCl_2_ 2.4 and KH_2_PO_4_ 1.2) at 37°C and at a constant pressure of 80 mmHg. According to the standard procedure in our laboratory (Hjortbak et al., 2018), hearts stabilized for 20 min before being subjected to 30 or 40 min of no‐flow global ischemia depending on the protocol. In our initial pilot studies, we found that Sprague–Dawley rats had a lower tolerance to ischemia (Figure 2). To bring the IS/AAR in the control group close to 50%, the protocol for the Sprague–Dawley animals was changed to 30 min global ischemia. This approach allows for a comparable infarct size modulation by IPC and RIC between strains. Reperfusion was initiated and continued for 120 min before the heart was removed from the setup, frozen, and stored at −80°C for later analysis.

Coronary flow (CF) was measured in the perfusion setup using an inline flowmeter (Hugo Sacs Electronic). During the entire protocol, a pressure transducer connected to a fluid‐filled balloon in the left ventricle monitored left ventricular function. Data were acquired with dedicated acquisition software (Notocord®) and stored on a PC for analysis. (Figure S1–S9).

### 2.3 Infarct size measurements

The frozen hearts were sliced and stained using a 1% Triphenyl Tetrazolium Chloride (TTC). After staining, the heart slices were stored in 4% formalin buffer (VWR International). After 24 h, the slices were weighed and scanned using a flatbed scanner (Epson Perfection V600 Photo scanner; Epson). The images were analyzed digitally using ImageJ (NIH) to evaluate infarct size and area at risk (IS/AAR).

### 2.4 Anesthesia

Depending on the protocol, the animals were sedated using two different regimens: (1) a mixture of Dormicum® (midazolam, 0,5 mg/kg; Matrix Pharmaceuticals), Hypnorm® (fentanylcitrate, 0,158 mg/kg and fluanisone 0,5 mg/kg; Vetapharma Ltd.) and sterile water (dosage 0.2 mL of mixture/100 g body mass) (rodent mixture), followed by supplementary injections of 40% of the initial dose every 30 min or (2) pentobarbiturate (65 mg/kg body weight; Skanderborg Pharmacy) followed by supplementary injections of 40% of the initial dose every 30 min. The rodent mixture was given subcutaneously, whereas pentobarbiturate was given intra‐peritoneally. When anesthesia was achieved, the animals were intubated and connected to a mechanical ventilator supplied with atmospheric air. To ensure a stable temperature (37°C ± 1°C) the animals were placed on a heating plate with a rectal thermometer (UNO, Zevenaar, Netherlands).

### 2.5 IPC and RIC protocols

**FIGURE 3:**
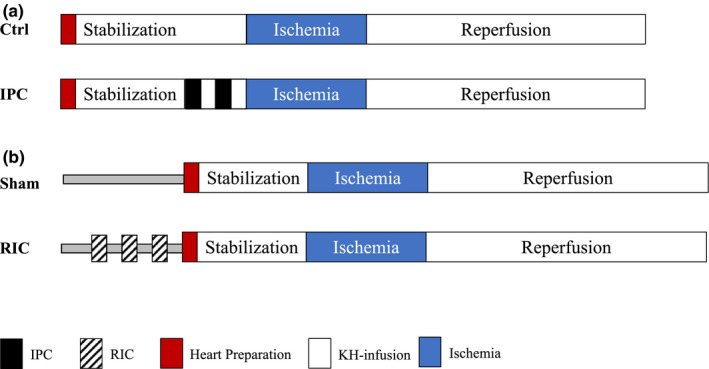
Graphical representation of the study protocols. a) protocols for IPC and corresponding control, b) protocols for RIC and corresponding Sham. IPC, Ischemic preconditioning; KH, Krebs‐Henseleit; RIC, Remote ischemic conditioning

We used similar protocols for IPC and RIC treated animals (Figure 3). IPC was induced by 2 × 5 min of global ischemia interrupted by 5 min reperfusion in the Langendorff system before the prolonged index ischemia. The control hearts were perfused in the Langendorff system without ischemia for the entire 20 min, prior to the prolonged ischemia. Importantly, the hearts from the IPC groups were removed immediately after anesthesia was achieved (Figure 3a). The RIC procedure was initiated 10 min after anesthesia was achieved and the animal was connected to the ventilator. The entire RIC procedure was performed in vivo, lasted a total of 30 min, and consisted of 3 × 5 min ischemia and 5 min reperfusion using a tourniquet on one hind limb. Sham animals were sedated and connected to a ventilator for 40 min, but were not subjected to the brief episodes of hindlimb IR. The RIC and sham hearts were removed and connected to the Langendorff perfusion setup after the in vivo procedure was concluded. During the RIC procedure, it was ensured that the tourniquet was sufficiently tightened to totally occlude blood flow during the ischemic period by the paling of the foot and subsequent hyperemia during reperfusion. In a small sub‐study, we confirmed the absence of blood flow in the hind limb of the animals, using a dedicated rodent ultrasound probe (VisualSonic) to measure flow in the arteries, before, during, and after tightening of the tourniquet (data not shown).

### 2.6 Statistical analyses

All data were analyzed using GraphPad Prism 8.2.0 (Graph Pad Software). All data were compared using the simple Student's t test for pairwise comparison and 2‐way ANOVA for comparison of more than two groups with subsequent pairwise comparison by post hoc LSD Fischer's test when appropriate. Results are presented as mean ± SD and a p value <0.05 was considered significant. The study design is explorative and therefore the required samples of size 8–10 rats were based on experience from previously published work using the isolated heart model (Hauerslev et al., 2018). The assumption allows an absolute reduction in infarct size by IPC and RIC of 17% and standard deviation of 11% with an alpha of 0.05% and a power of 80%.

## 3 RESULTS

### 3.1 Ischemia time

Global ischemia of 40 min resulted in larger infarct sizes than 30 min global ischemia in Sprague–Dawley rats (Figure 2). IPC induced infarct size reduction with both 30 (48.4 ± 18 vs. 20.3 ± 7, *p* < 0.001) and 40 min of ischemia (78.8 ± 9 vs. 58.3 ± 12, *p* < 0.01).

### 3.2 Strain

**FIGURE 4:**
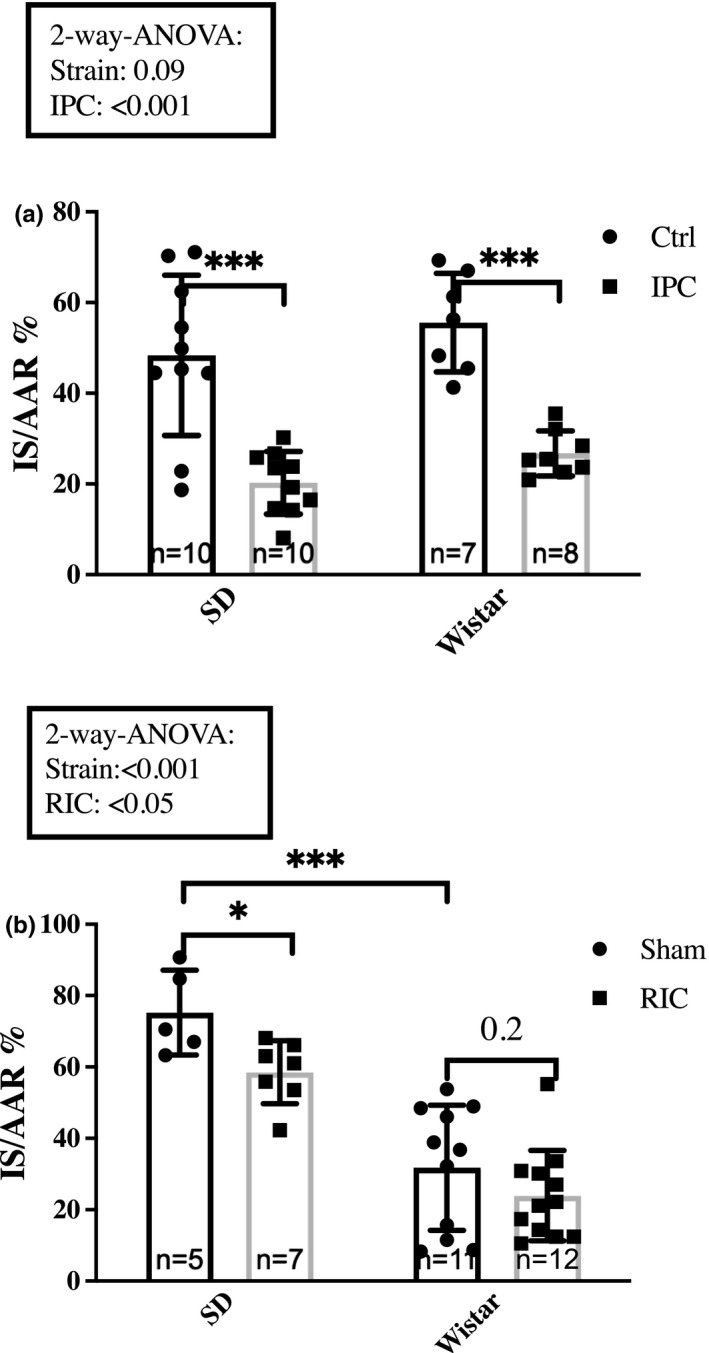
Impact of strain. The impact of strain on infarct size attenuation by (a) IPC and (b) RIC in 7‐week Sprague–Dawley and Wistar rats, respectively. Rats were sedated with rodent mixture. Mean ± SD *p < 0.05, **p < 0.01 and ***p < 0.001. Ctrl, Control; IPC, Ischemic preconditioning; IS/AAR, infarct size/Area at risk; RIC, Remote ischemic conditioning; SD, Sprague–Dawley

The effect of IPC was investigated in 7‐weeks‐old Sprague–Dawley and Wistar rats from Taconic. Two‐way ANOVA analysis revealed no statistically significant difference between strains on infarct size (*p* = 0.09). The mean infarct size among Sprague–Dawley rats was larger in the Sham group than in the control group (75.3 ± 11.8% vs. 48.4 ± 17.7%, *p* < 0.01) (Figure 4a+b), whereas the opposite was observed in Wistar rats (55.6 ± 10.9% vs. 31.8 ± 17.6, *p* < 0.01) (Figure 4 a + b).

IPC attenuated infarct sizes in both Sprague–Dawley rats (48.4 ± 17.7% vs. 20.3 ± 6.9, *p* < 0.001) and Wistar rats (55.6 ± 10.9% vs. 26.8 ± 5.0%, *p* < 0.001) (Figure 4a), corresponding to 58% and 52% relative infarct size reduction, respectively.

The effect of RIC was studied in 7‐weeks‐old Sprague–Dawley and Wistar rats from Taconic. Two‐way ANOVA analysis revealed a difference in infarct size between strains as well as an effect of RIC on infarct size (*p* < 0.001 and *p* < 0.05, respectively) (Figure 4b). Subsequent post hoc pairwise comparison demonstrated that infarct sizes in the sham groups were larger in the Sprague–Dawley rats than in the Wistar rats (75.3 ± 11.8% vs. 31.8 ± 17.6%, *p* < 0.001). Moreover, RIC induced a statistically significant decrease of infarct size in Sprague–Dawley rats (75.3 ± 11.8% vs. 58.6 ± 8.9%, *p* < 0.05) corresponding to a 22% relative reduction. In Wistar rats, infarct size also decreased corresponding to a 24% relative infarct size reduction but the reduction was not statistically significant (31.7 ± 17.6% and 24.0 ± 12.6%, *p* = 0.2), (Figure 4b).

### 3.3 Age

**FIGURE 5:**
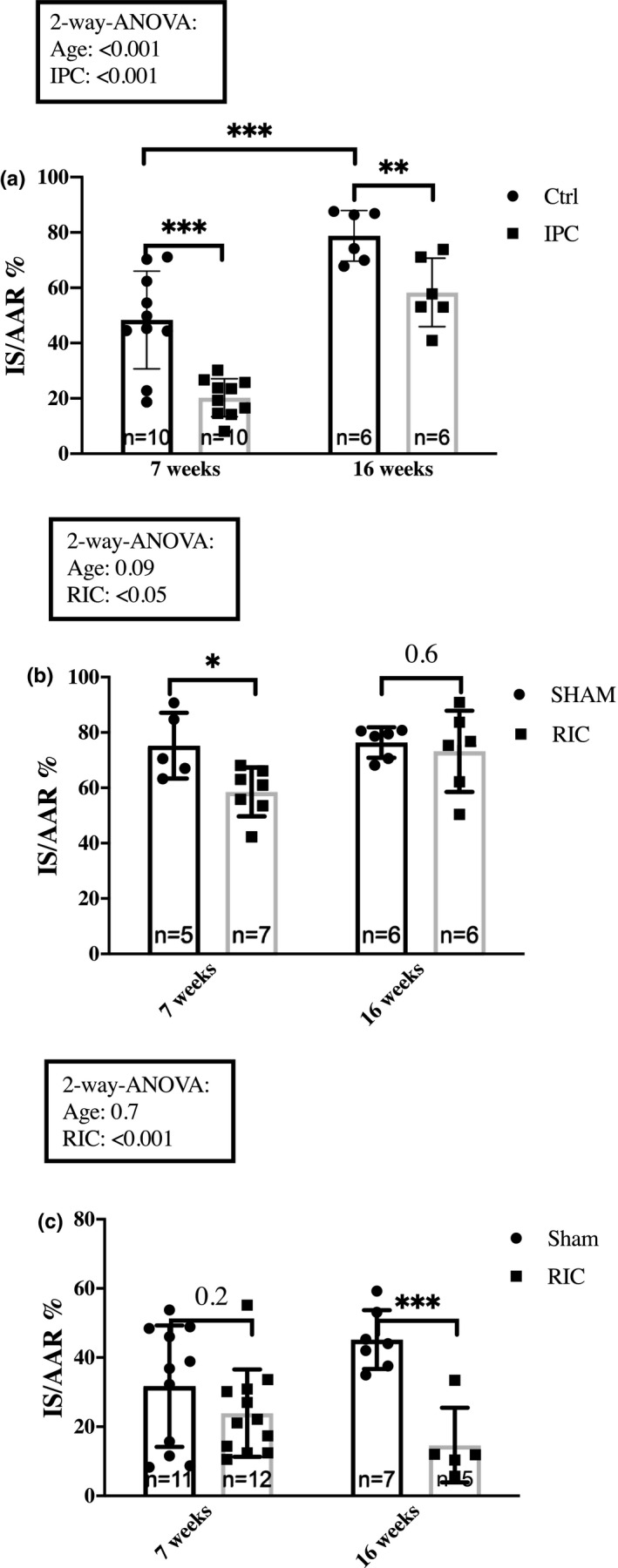
Impact of age. The impact of age on infarct size attenuation by (a) IPC in Sprague–Dawley rats, (b) RIC in Sprague–Dawley and (c) RIC in Wistar rats. Rats were sedated with rodent mixture. Mean ± SD. *p < 0.05, **p < 0.01 and ***p < 0.001. Ctrl, Control; IPC, Ischemic preconditioning; IS/AAR, infarct size/Area at risk; RIC, Remote ischemic conditioning

The effect of IPC on infarct size was investigated in 7‐ and 16‐weeks‐old Sprague–Dawley rats from Taconic. Two‐way ANOVA analysis demonstrated that both age and IPC modified infarct size (*p* < 0.001 and *p* < 0.001, respectively). Infarct sizes were significantly larger in 16‐weeks‐old Sprague–Dawley rats than in 7‐weeks‐old Sprague–Dawley rats in controls (78.8 ± 9.2% vs. 48.4 ± 17.7%, *p* < 0.001) and IPC reduced infarct sizes in both age groups (48.4 ± 17.7% vs. 20.3 ± 6.9%, *p* < 0.001 and 78.8 ± 9.2% vs. 58.3 ± 12.3%, *p* < 0.01) (Figure 5a).

We compared the effect of age on infarct size between Sprague–Dawley and Wistar rats (Figure 5b+c). Infarct sizes were also significantly higher in Sprague–Dawley than in Wistar rats at 16 weeks (76.4 ± 5.5% vs. 45.2 ± 8.5%, *p* < 0.001).

RIC reduced infarct size in 7‐weeks‐old Sprague–Dawley rats; we found no infarct size reduction in 16‐weeks‐old Sprague–Dawley rats (76.4 ± 5.5% and 73.2 ± 14.7%, *p* = 0.6) (Figure 5b). In contrast, RIC reduced infarct size in 16‐weeks‐old Wistar rats (45.2 ± 8.5% vs. 14.7 ± 10.8%, *p* < 0.001) (Figure 5c), but not in 7‐weeks‐old Wistar rats (*p* = 0.2).

### 3.4 Supplier

**FIGURE 6:**
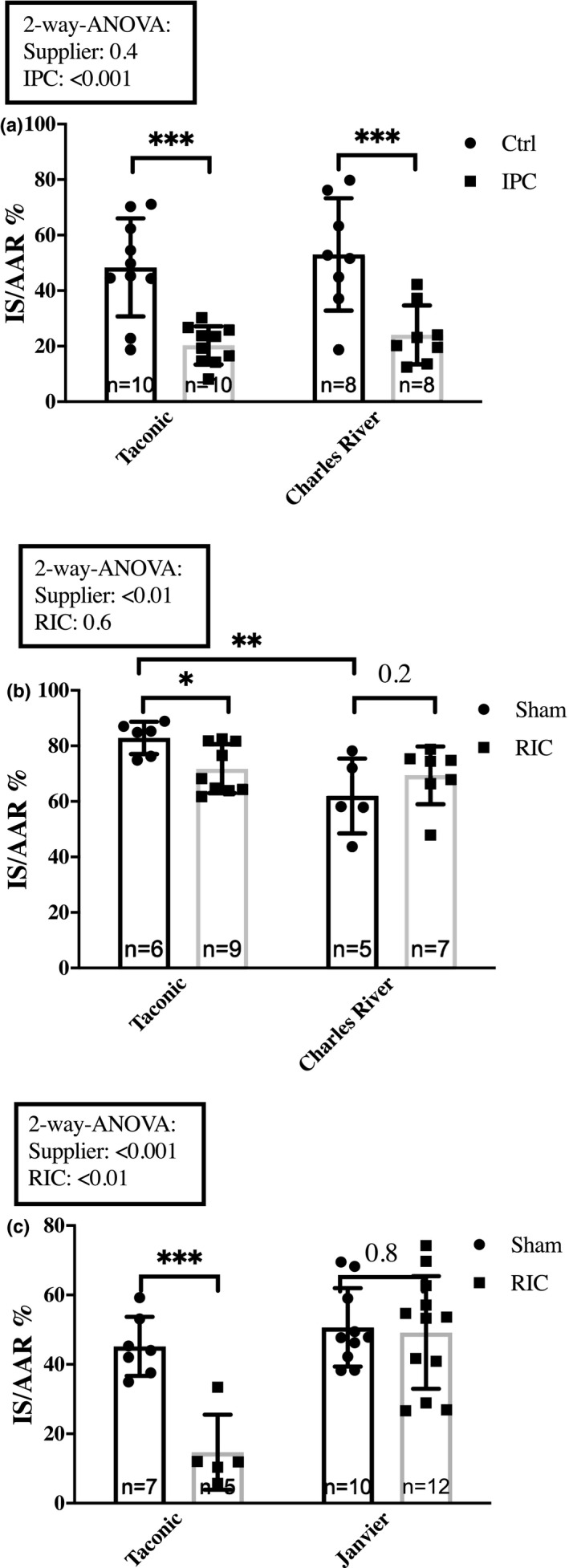
Impact of the supplier. The impact of the supplier on infarct size attenuation by (a) IPC in 7‐weeks‐old Sprague–Dawley rats from Taconic and Charles River, sedated with rodent mixture. (b) RIC in 7‐weeks‐old Sprague–Dawley rats from Taconic and Charles River, sedated with pentobarbiturate. (c) Comparison of RIC in 16‐weeks‐old Wistar rats from Taconic and Janvier, sedated with rodent mixture. Mean ± SD. *p < 0.05, **p < 0.01 and ***p < 0.001. Ctrl, Control; IPC, Ischemic preconditioning; IS/AAR, infarct size/Area at risk; RIC, Remote ischemic conditioning

We studied the impact of the supplier on infarct size in 7‐weeks‐old Sprague–Dawley rats from Taconic and Charles River. Two‐way ANOVA analysis revealed no effect of the supplier on the infarct size (*p* = 0.4), and infarct size reduction by IPC was similar (48.4 ± 17.7% vs. 20.3 ± 6.9%, *p* < 0.001; 53.0 ± 20.2% and 24.1 ± 10.6%, *p* < 0.001, respectively) (Figure 6a).

Comparison of RIC efficacy in the 7‐weeks‐old Sprague–Dawley rats from Taconic and Charles River demonstrated an effect of the supplier but not RIC on the infarct sizes (*p* < 0.01 and *p* = 0.6, respectively) (Figure 6b). Infarct sizes in the Sham groups were significantly larger in the Taconic rats than in the Charles River rats (82.9 ± 5.9% vs. 62.0 ± 13.5%, *p* < 0.01). We observed that RIC induced a statistically significant reduction of infarct size in the animals from Taconic (82.9 ± 5.9% vs. 71.7 ± 8.8%, *p* < 0.05), but not in the animals from Charles River (62.0 ± 13.5% and 69.4 ± 10.4% *p* = 0.2) (Figure 6b).

In 16‐weeks‐old Wistar rats from Taconic and Janvier, two‐way ANOVA analysis demonstrated an effect of both supplier and RIC on the results (*p* < 0.001 and *p* < 0.01, respectively). RIC reduced infarct size in rats from Taconic (45.2 ± 8.5% vs. 14.7 ± 10.8%, *p* < 0.001), but not in rats from Janvier (50.7 ± 11.3 and 49.2 ± 16.2, *p* = 0.8) (Figure 6c).

### 3.5 Anesthesia

**FIGURE 7:**
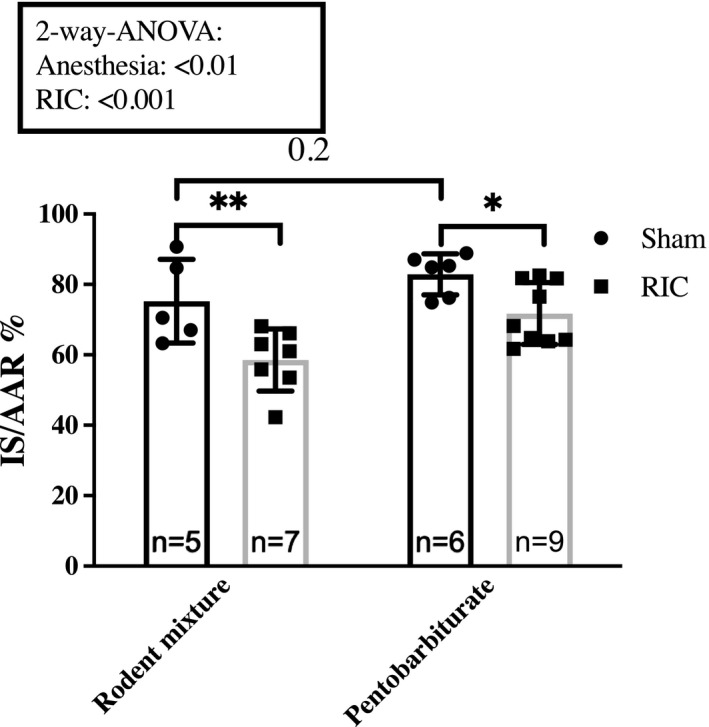
Impact of anesthesia. Comparison of infarct size attenuation by RIC between 7‐weeks‐old Sprague–Dawley rats sedated with rodent mixture and Pentobarbiturate. Mean ± SD. **p < 0.05 and **p < 0.01. IS/AAR, infarct size/Area at risk; RIC, Remote ischemic conditioning

We compared anesthesia in 7‐week‐old Sprague–Dawley rats. We found no difference in infarct size between rats sedated with rodent mixture or pentobarbiturate in the Sham groups (75.2 ± 11.8% and 82.9 ± 5.9%, *p* = 0.2). RIC reduced infarct size with rodent mixture and pentobarbiturate (rodent mixture: 75.2 ± 11.8% vs. 58.6 ± 8.9%, *p* < 0.01; pentobarbiturate: 82.9 ± 5.9% and 71.7 ± 8.8%, *p* < 0.05) (Figure 7).

## 4 DISCUSSION

Our main findings are that the sensitivity of IR‐injury and the response to conditioning by IPC were consistent, while the efficacy of RIC varies between rat strains when studied in an ex vivo model. Additionally, age, anesthesia, supplier of the animals, and preoperative animal care influenced sensitivity to IR‐injury and response to RIC. These findings demonstrate that standardized recommendations for consistency are needed to ensure comparability and reproducibility in preclinical ex vivo studies on cardioprotection between experimental laboratories.

When we compared infarct size in 7‐weeks‐old Sprague–Dawley and Wistar rats from the same supplier (Taconic), sedated with the rodent mixture, our results reflected that sensitivity to IR‐injury was higher in Sprague–Dawley than in Wistar rats. Applying 40 min of ischemia yielded significantly larger infarct size in Sprague–Dawley than in Wistar rats. To compare the response to IPC and RIC at infarct sizes of similar magnitude, we, therefore, chose to reduce ischemia time in the Sprague–Dawley rats from 40 to 30 min. This duration of ischemia yielded an infarct size of 50% of the left ventricle. Although the infarct size is much larger than those registered in a clinical setting of myocardial infarction,(Bøtker et al., 2010; Hausenloy et al., 2019) this order of magnitude allows for the detection of infarct size reduction in proof‐of‐concept studies of cardioprotection by a variety of pharmacological and mechanical interventions and may also leave room for the detection of any harmful effects that might increase infarct size. The infarct size was similar to the size of the infarctobtained in Wistar rats with 40 min of ischemia in our global ischemia ex vivo setting when IPC was induced in the Langendorff system. Further evidence a high sensitivity to IR‐injury in Sprague–Dawley rats was obtained by our findings that infarct size increased significantly in this strain when the Sham rats in the RIC setting were exposed to a 40 min preischemic period that was needed to allow for identical study settings with the intervention group. This led to larger infarct sizes in the Sham group than in the control group. In contrast, the infarct size decreased in the Wistar rats, demonstrating a very different response to the preischemic in vivo handling and prolonged anesthetic period that seemed to aggravate an ischemic response in Sprague–Dawley rats but rather induce cardioprotection in Wistar rats.

The mechanisms underlying this opposing behavior most likely involve genetic components responsible for resistance to myocardial ischemia (Baker et al., 2000). A comparison of infarct size in isolated hearts from inbred and outbred rats demonstrated highly variable sensitivity to ischemia and reperfusion (Baker et al., 2000). Because any difference in external factors such as providers, chow, and animal handling was minimized, assessment of strain relatedness identified genetic components to be responsible for the sensitivity to myocardial IR‐injury (Baker et al., 2000). Further support may be gained from studies of renal ischemia and reperfusion, which have demonstrated that leucocyte infiltration and major histocompatibility complex (MHC) class II expression was less in PVG and Wistar than in Lewis and Dark Agouti rats, and that the response depended on genetic differences in MHC class II expression (Ibrahim et al., 1996). Extrapolating to the heart, interstitial cell expression of MHC class II expression in rat hearts is also under genetic control (Darden et al., 1990), suggesting that similar mechanisms may prevail in the heart. Although we did not investigate genetic involvement, the response to cardioprotective strategies, like IPC and RIC, which modulate the inflammatory response to IR‐injury, may be genetically determined (Albrecht et al., 2013; Konstantinov et al., 2004; Shimizu et al., 2010). The comparison between strains and simultaneous use of molecular genetics may allow the localization of gene(s) and promote mechanistic insight in mechanisms responsible for sensitivity to myocardial IR‐injury.

With the large infarct sizes that we obtained in 7‐weeks‐old Sprague–Dawley rats, we were able to demonstrate a statistically significant reduction in infarct size by IPC as well as RIC. Translated into relative reductions the infarct sizes were reduced corresponding to 58% and 22% by IPC and RIC, respectively, indicating that IPC seems to be a more potent cardioprotective modality than RIC (Botker, Lassen, et al., 2018). From a translational perspective, the infarct size with modern reperfusion therapy in current clinical practice is 16% of the left ventricle (Bøtker et al., 2010; Hausenloy et al., 2019), so an infarct size of this magnitude might be an optimal target in experimental models.

While the reduction of infarct size by IPC in Wistar rats was in the same order of magnitude as Sprague–Dawley rats, the reduction by RIC did not achieve statistical significance in Wistar rats. These findings seem to indicate that cardioprotection by RIC is achievable in Wistar rats. However, a large number of rats are needed due to variability. In our experience, group sizes of 8–10 animals are usually required to obtain statistically valid results concerning infarct size reduction in experimental ex vivo proof‐of‐concept studies (Hauerslev et al., 2018).

Age is a well‐known confounder of the efficacy of IPC (Boengler et al., 2009; Calabrese, 2016) and RIC (Behmenburg et al., 2017). We found that 16‐weeks‐old rats had larger infarcts than 7‐weeks‐old rats, but only in the IPC protocol. However, in the RIC protocol, age did not have an effect on the cardioprotective capacity. Lu et al. demonstrated that the protection by IPC was attenuated in 6‐months‐old Sprague–Dawley rats compared to 2‐months‐old rats (Lu et al., 2001). A similar decrease in the protection offered by IPC (Abete et al., 1996) and RIC (Behmenburg et al., 2017) related to older age has also been demonstrated in Wistar rats although the animals used were much older than the animals used in our study (20–24 months). The discrepancy is most likely explained by the fact that 16‐weeks‐old animals used in our study cannot be considered old.

The response to RIC seemed to be dependent of the supplier. The differences may be caused by dissimilarities in environmental factors between each supplier (Hübinette et al., 2001). However, the differences may also be due to genetic drift in the outbreeding program of the rats (Festing, 1993) or differences in the rats’ gut microbiome (Tibbs et al., 2019). Regardless of the cause, the different responses to RIC in rats from different suppliers should be kept in mind when comparing findings from different laboratories with different suppliers. Interestingly, the IPC intervention did not seem to be affected by the supplier, which may further support the notion that IPC is a more robust modality than RIC. This is also in accordance with the documented ability of IPC to induce cardioprotection in humans during coronary intervention (Deutsch et al., 1990) and CABG (Yellon & Davidson, 2014) and the difficulty in successfully translating RIC to the clinic (Hausenloy et al., 2019).

Many studies have demonstrated that drugs for the sedation of animals pose a major influence on the efficacy of IPC and RIC (Bunte et al., 2019). Propofol has most consistently been associated with reduced response to RIC, possibly due to a decreased release of humoral factors (Bunte et al., 2019; Hausenloy et al., 2015; Kottenberg et al., ,,^2012^, ^2014^). In contrast, sedation with volatile anesthetics like isoflurane activates the same cardioprotective pathways associated with the cardioprotection induced by IPC and RIC (Cason et al., 1997; Kersten et al., 1997). Pentobarbiturate has a negative inotropic effect and causes hypotension due to peripheral vasodilation (Tobias & Leder, 2011). We found no difference between the anesthetic regimes in our study. We did not measure blood pressure or other invasive measures during the RIC intervention and therefore we are unable to identify potential differences in hemodynamics between the anesthetic regimens.

We based our algorithm of RIC using three cycles on one hind limb on previous results (Johnsen et al., 2016). The number of cycles may differ between the two species and the optimal RIC protocol in rats has not been documented. However, our data demonstrated that the applied RIC protocol yielded consistent efficacy in different rat strains. The metanalysis by Bromage et al. did not disclose a difference in infarct size between the number of cycles (between 1 and 4), between the duration of occlusion (5–15 min) or between the number of limbs (1 or 2) in an in vivo model (Bromage et al., 2017).

Our rats were ventilated with atmospheric air. We intentionally chose this approach to avoid adding oxygen because a study by Davidson et al. demonstrated that oxygen is a confounder of RIC efficacy in mice (Davidson et al., 2017).

We used only male rats to avoid interference from fluctuation in female sex hormones. Experimental observations have confirmed the results of epidemiological studies investigating sex‐specific differences in cardiac tolerance to ischemia (Ostadal & Ostadal, 2014). The IR‐injury is diminished in pre‐menopausal female rats compared to age‐matched male rats (Ostadal & Ostadal, 2014). Detailed mechanisms of sex‐related differences remain unknown and may involve genomic and non‐genomic effects of sex steroid hormones, particularly the estrogens, which have been the most extensively studied (Botker et al., 2020; Fels & Manfredi, 2019). Future studies are needed to study the impact of sex on IR‐injury and the potential for modulation (Perrino et al., 2020).

The meta‐analysis of RIC by Bromage et al. in in vivo models revealed a rather consistent positive effect of RIC, as only one study in the meta‐analysis did not achieve protection by RIC. Preischemic RIC in the in vivo studies reached an average of 23% point IS/AAR reduction, which is comparable to our findings in the ex vivo model. However, RIC in our ex vivo experiments did not seem to ensure consistent protection in all settings, suggesting that the ex vivo model may be less suitable for revealing the full cardioprotective potential by RIC due to a lack of inherent whole‐body signaling pathways. In in vivo as well as ex vivo studies, a detailed characterization of the RIC procedure is mandatory for comparison purposes between studies. As recently proposed for cardioprotection studies in large animal models (Rossello et al., 2019), a standardized platform for the experimental settings is highly recommendable for RIC research in rodents.

There are several limitations to our study. First, our study was designed as an explorative study to identify the optimal setting for the study of IPC and RIC in rats in our laboratory, and underlying mechanisms were not sought. Limitations attributed to the experimental setup include the use of global ischemia, which contrasts the regional ischemia seen in myocardial infarction in humans. The IPC stimulus is applied during perfusion in the Langendorff set‐up, whereas the RIC stimulus is applied before the isolation of the heart and requires a prolonged anesthetic period that may influence infarct size. The various responses in infarct size between Sprague–Dawley and Wistar rats suggests that the influence of such confounders may vary between strain depending on the sensitivity to IR‐injury.

Because of the explorative design of our study, the data set is not completely systematic. As a consequence, the comparison of ischemia time is also a comparison between different aged rats. Additionally, we frequently chose to pursue a specific direction based on our results. We were unable to add additional Wistar rats to the groups, because the supplier (Taconic) discontinued the production of the strain.

In conclusion, pre‐clinical ex vivo studies of myocardial IR‐injury document a significant and highly reproducible beneficial effect of IPC. RIC did not produce a similar strong stimulus, but still yields protection across different strains. Age, animal supplier, and anesthetics may modulate the sensitivity of IR‐injury and the response to RIC. Based on our findings a standardized experimental platform is recommended to ensure consistency and reproducibility of preclinical studies of cardioprotective interventions in rodents.

## CONFLICT OF INTEREST

None to declare.
